# Mortality prediction by bispectral electroencephalography among 502 patients: its role in dementia

**DOI:** 10.1093/braincomms/fcab037

**Published:** 2021-03-13

**Authors:** Taku Saito, Johnny R Malicoat, Lydia R Leyden, Jessica C Williams, Sydney S Jellison, Hailey Long, Mandy M Hellman, Kaitlyn J Crutchley, Zoe-Ella E M Anderson, Duachee Lo, Manisha V Modukuri, Cassidy J Schacher, Aihide Yoshino, Hiroyuki Toda, Eri Shinozaki, Hyunkeun R Cho, Sangil Lee, Gen Shinozaki

**Affiliations:** 1 Department of Psychiatry, University of Iowa, Iowa City, IA 52242, USA; 2 Department of Psychiatry, School of Medicine, National Defense Medical College, Tokorozawa, Saitama 359-8513, Japan; 3 Department of Internal Medicine, University of Iowa, Iowa City, IA 52242, USA; 4 Department of Biostatistics, College of Public Health, University of Iowa, Iowa City, IA 52242, USA; 5 Department of Emergency Medicine, University of Iowa, Iowa City, IA 52242, USA; 6 Department of Neurosurgery, University of Iowa, Iowa City, IA 52242, USA; 7 Department of Anesthesia, Carver College of Medicine, University of Iowa, Iowa City, IA 52242, USA; 8 Iowa Neuroscience Institute, University of Iowa, Iowa City, IA 52242, USA; 9 Interdisciplinary Graduate Program in Neuroscience, University of Iowa, Iowa City, IA 52242, USA

**Keywords:** delirium, dementia, bispectral electroencephalogram, BSEEG, mortality

## Abstract

Complications of delirium and dementia increase mortality; however, it is difficult to diagnose delirium accurately, especially among dementia patients. The bispectral electroencephalography score can detect delirium and predict mortality in elderly patients. We aimed to develop an efficient and reliable bispectral electroencephalography device for high-throughput screening. We also hypothesized that bispectral electroencephalography score can predict mortality among dementia patients. A prospective cohort study was conducted between January 2016 and December 2018 to measure bispectral electroencephalography from elderly patients and correlate with outcomes. A total of 502 elderly (55 years old or older) patients with and without dementia were enrolled. For a replication of the utility of bispectral electroencephalography, mortalities between bispectral electroencephalography-positive and bispectral electroencephalography-negative group were compared. In addition, patients with and without dementia status were added to examine the utility of bispectral electroencephalography among dementia patients. The mortality within 180 days in the bispectral electroencephalography-positive group was higher than that of the bispectral electroencephalography-negative group in both the replication and the total cohorts. Mortality of those in the bispectral electroencephalography-positive group showed a dose-dependent increase in both cohorts. When the dementia patients showed bispectral electroencephalography positive, their mortality was significantly higher than those with dementia but who were bispectral electroencephalography-negative. Mortality within 30 days in the bispectral electroencephalography-positive group was significantly higher than that of the bispectral electroencephalography-negative group. The utility of the bispectral electroencephalography to predict mortality among large sample of 502 elderly patients was shown. The bispectral electroencephalography score can predict mortality among elderly patients in general, and even among dementia patients, as soon as 30 days.

## Teaser

Saito et al. aimed to show that their bispectral electroencephalography (BSEEG) method can capture brain wave abnormalities indicative of delirium and predict mortality among elderly patients with and without dementia. They previously showed the usefulness of BSEEG with 274 inpatients, and this time they studied an additional 228 subjects to replicate their findings.

## Graphical Abstract

**Graphical Abstract:**
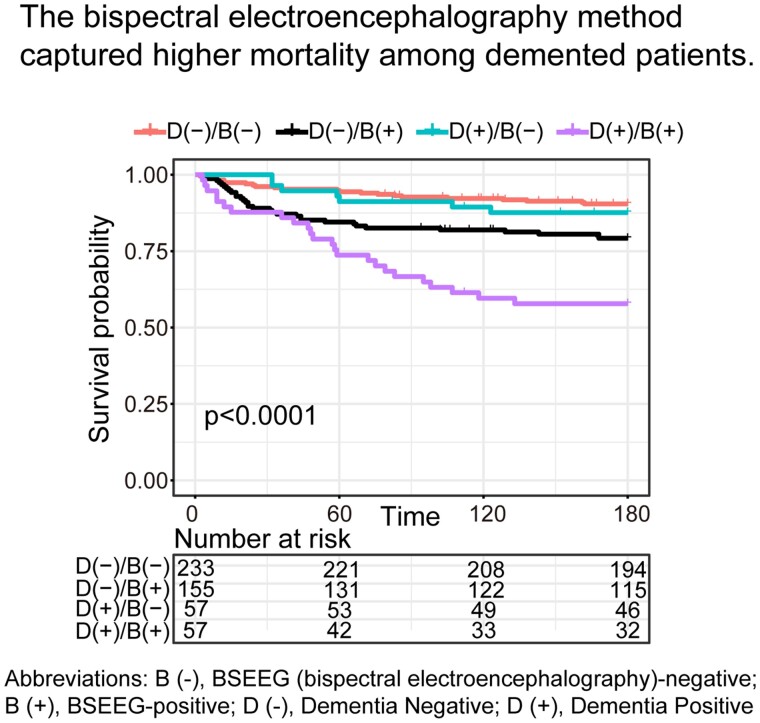

## Introduction

The relationship between delirium and dementia is complicated because dementia is one of the risk factors of delirium.^1^^,^^2^ In addition, delirium is known to accelerate the progression of dementia.^2^ Furthermore, delirium and dementia are associated with patients’ outcomes including mortality.^3^ Especially if patients have both delirium and dementia, their mortality would increase.^4^^,^^5^

Although it is important to diagnose delirium accurately for prompt intervention in the elderly, it is often difficult.^6^^,^^7^ We previously developed a method to detect delirium by using the bispectral electroencephalography (BSEEG) score.^7^^,^^8^ Higher BSEEG scores were associated with higher mortality independent of delirium status.^9^ In fact, we showed that BSEEG score can predict mortality of elderly inpatients better than clinical categorization of delirium.^9^ Consistent with our previous findings of higher BSEEG score indicative of slow brain wave and its association with increased mortality, a recent prospective cohort study^10^ and a retrospective cohort study^11^ showed that clinical electroencephalography slowing is associated with higher mortality. However, there is neither a replication study for the utility of BSEEG nor an investigation of the utility of BSEEG for subjects with dementia. Without detecting delirium, patients remain at high risk for poor outcomes including mortality.^4^^,^^12^

Therefore, first we conducted a replication study to confirm the utility of BSEEG. Next, we hypothesized that BSEEG score can predict mortality among dementia patients. To examine this hypothesis, we compared mortality in dementia patients by dividing subjects into two groups: Those with high and low BSEEG scores.

## Materials and methods

### Participants

We expanded the original prospective cohort study as reported previously.^9^ Briefly, patients were recruited from January 2016 to December 2018 in general medicine and orthopaedic services in the University of Iowa Hospitals and Clinics. Written informed consent was acquired from all participants or from their legally authorized representatives in cases in which patients were judged to be delirious or demented and lacked capacity to consent. In the present study, 228 patients (replication cohort) were selected to be consistent with the 274 subjects (discovery cohort) from the original study.^9^ These were a convenience sample. The participants of the discovery cohort were recruited from January 2016 to October 2017, and the replication cohort was from the additional cohort recruited up to December 2018. Both cohorts were enrolled in a similar way following the same protocol from January 2016 to December 2018 who aged 55 years or older. Taken together, 502 patients (discovery and replication cohorts) were subjected to analysis in the present study. All subjects provided written informed consent after receiving a complete description of the study. This research was approved by the University of Iowa Human Subjects Research Institutional Review Board and carried out in accordance with the Declaration of Helsinki.

### Clinical outcomes

The details about delirium status definition were described previously.^9^ Briefly, we screened for delirium by using the following: The Confusion Assessment Method for the Intensive Care Unit,^13^^,^^14^ the Delirium Rating Scale-Revised-98^15^ and the Delirium Observation Screening Scale.^16^ Delirium status was defined according to the results of the following screening tests: Confusion Assessment Method for the Intensive Care Unit positive, Delirium Rating Scale-Revised-98 score ≥19 or Delirium Observation Screening Scale score ≥3. Baseline cognitive function was measured by using the Montreal Cognitive Assessment^17^ when it was possible based on patients’ capacity and willingness to administer. Dementia was recorded based on chart review. Delirium and dementia statuses were finally determined by a board-certified consultation-liaison psychiatrist (G.S.) with the results of the measures and detailed chart review. Mortality status information was identified by electronic medical record review and/or obituary record. We have assessed mortality status up to 180 days for 100% of the 502 enrolled participants.

### BSEEG data collection and score definition

Details about BSEEG data collection and score definition have been described previously.^7^^,^^9^ Briefly, BSEEG data were collected by using a portable electroencephalography device (CMS2100, CONTEC, Qinhuangdao, Hebei, China) by trained research assistants twice daily at the same time of the clinical symptom assessment. One electrode was placed on the centre of patients’ forehead as a ground, and two electrodes were placed on the left and right sides of the forehead in case of two-channel recording, and one electrode was placed on the one side of the forehead in case of one-channel recording to obtain BSEEG signals for up to 10 min. While recording data, patients were instructed to keep their eyes closed and jaw relaxed. The obtained data were converted into spectral density plots, and a BSEEG score was produced by using the signal-processing algorithm. The cut-off score used was BSEEG score = 0 as reported in the previous study.^9^

### Statistical analysis

All statistical analyses were conducted using R.^18^ A *t*-test was conducted to compare continuous data, and a chi-square test or a Fisher's exact test was conducted to compare categorical data between cases and controls for delirium and dementia, and positive and negative for BSEEG. A log-rank test was conducted to compare two survival functions in 180 days. Moreover, mortality of both BSEEG-positive and -negative groups at the time of 30 days was compared to test how soon BSEEG can differentiate mortality risk. In addition, relative risk of the mortality in 30 days was calculated between the BSEEG-positive and -negative groups. Cox proportional hazards regression analysis was conducted to calculate the hazard ratio adjusting age, sex and the Charlson Comorbidity Index (CCI).^19^ A *P-*value of <0.05 was determined to be statistically significant.

### Data availability

The data will be shared by the corresponding author upon request with a reasonable institutional approval.

## Results

### Replication of the utility of BSEEG in prediction of mortality

**Figure 1:**
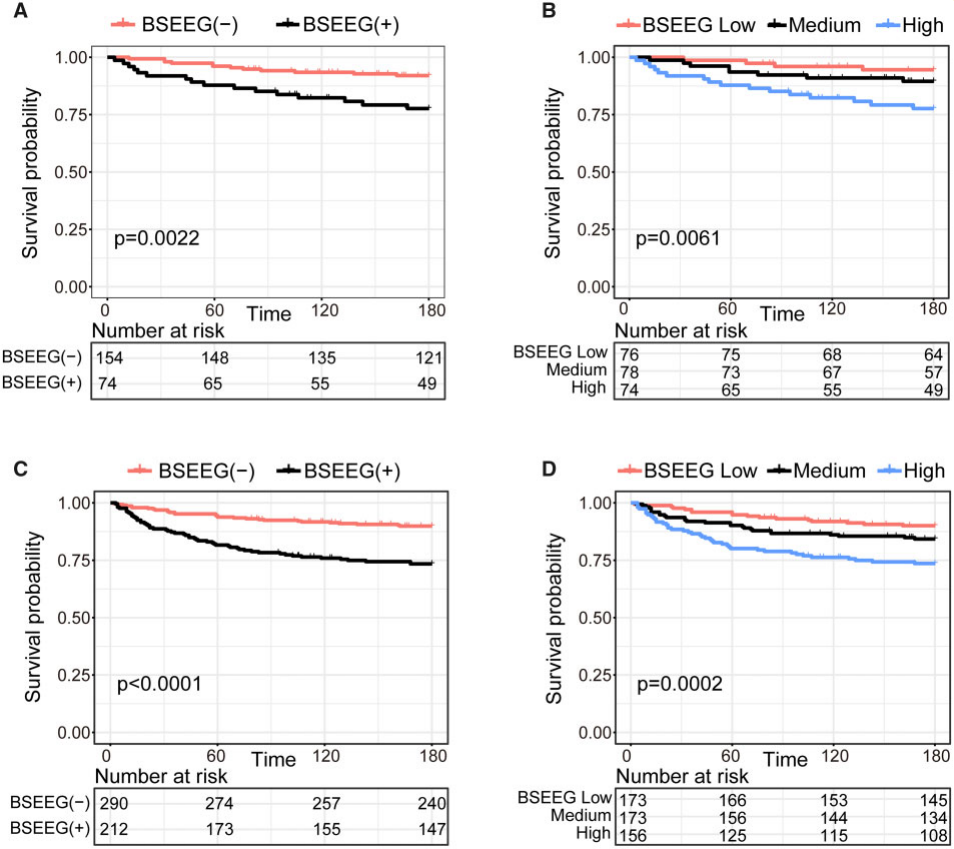
**Survival curve in 180 days based on the BSEEG category.** (**A**) Two BSEEG categories in 228 subjects (replication cohort). (**B**) Three BSEEG categories in 228 subjects (replication cohort). (**C**) Two BSEEG categories in 502 subjects (discovery and replication cohorts). (**D**) Three BSEEG categories in 502 subjects (discovery and replication cohorts). BSEEG, bispectral electroencephalography; B (-), BSEEG-negative; B (+), BSEEG-positive.

We first analysed 228 subjects (replication cohort) for a replication study to confirm the utility of BSEEG in prediction of mortality. Their demographic characteristics are shown in Supplementary Table 1. Age and CCI were significantly higher in patients with delirium, dementia and BSEEG-positive groups, compared to each of the control groups (Supplementary Table 1). The proportion of female subjects was significantly higher in patients with dementia compared to the control group (Supplementary Table 1). The unadjusted mortality in 180 days in the BSEEG-positive group was higher than that of the BSEEG-negative group (Fig. 1A). When the patients were divided into three categories to become approximately equal sample sizes with BSEEG low, medium and high based on BSEEG scores, their mortality showed a dose-dependent increase based on the BSEEG categories (Fig. 1B). According to the result of the Cox proportional hazard model adjusted for age, sex, CCI and delirium, BSEEG score was shown to be a significant predictive factor for mortality in 180 days (95% confidence interval, 1.33–6.00; *P* = 0.007) (Supplementary Table 2). In addition, age and CCI were significant predictors for mortality (Supplementary Table 2).

Next, we analysed 502 subjects (discovery and replication cohorts). Their demographic characteristics are shown in Table 1. The subjects were recruited from the following units: 273 (54.4%) were general medicine, 141 (28.1%) were orthopaedics, 66 (13.2%) were emergency department and 22 (4.4%) were intensive care unit. The average number of the BSEEG recordings was 4.1 ± 3.3. Age and CCI were significantly higher in patients with delirium, dementia and in BSEEG-positive groups, compared to respective control groups (Table 1). The unadjusted mortality in 180 days in the BSEEG-positive group was higher than that of the BSEEG-negative group (Fig. 1C). Moreover, when the patients were divided into three categories to become approximately equal sample sizes with BSEEG low, medium and high based on the BSEEG scores, their mortality showed a score-dependent increase based on the BSEEG categories (Fig. 1D). According to results of the Cox proportional hazard model adjusted for age, sex, CCI and delirium, BSEEG showed significant predictive factor for mortality in 180 days (95% confidence interval, 1.55–3.82; *P* < 0.001) (Supplementary Table 3). Age, delirium status and CCI were significant predictors for mortality (Supplementary Table 3).

**Table 1:** Demographic characteristic of the discovery and replication cohorts (*N* = 502)

	Delirium		Dementia		Day1 BSEEG	
	Case	Control	Case	Control	Positive	Negative
n	168	334	114	388	212	290
Female, *n* (%)	81 (48.2)	177 (53.0)	68 (59.6)	190 (49.0)	106 (50.0)	152 (52.4)
Age, mean (SD), (year)	73.5 (9.4)^*^	71.6 (9.8)	76.3 (9.0)^*^	71.0 (9.6)	72.8 (9.0)^*^	71.8 (10.2)
CCI, mean (SD)	4.3 (2.8)^*^	3.0 (2.9)	4.3 (3.0)^*^	3.1 (2.9)	3.9 (2.9)^*^	3.0 (2.9)

* *P* < 0.01 vs. Control or Negative.

BSEEG, bispectral electroencephalography; CCI, Charlson Comorbidity Index.

### Utility of BSEEG in predicting mortality among patients with and without dementia

**Figure 2:**
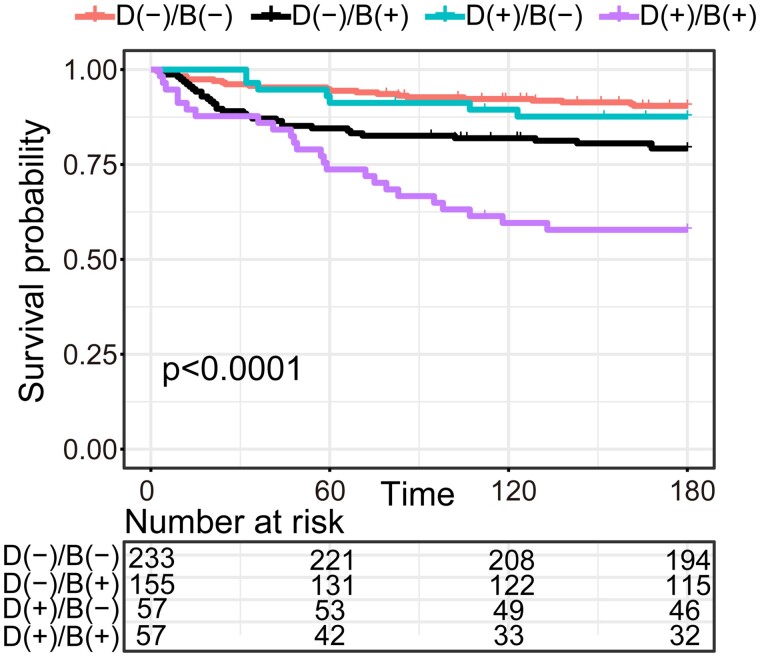
**Survival curve in 180 days based on the dementia and BSEEG categories.** BSEEG, bispectral electroencephalography; B (-), BSEEG-negative; B (+), BSEEG-positive; D (-), Dementia Negative; D (+), Dementia Positive.

Next, we analysed the total 502 subjects (discovery and replication cohorts) to test the utility of BSEEG for predicting mortality in patients with dementia. When patients with dementia were shown to be BSEEG-positive, their mortality was higher than those with dementia but who were BSEEG-negative (Fig. 2). When dementia was added as a covariate in the Cox proportional hazard model, BSEEG was still shown to be a significant predictive factor for mortality in 180 days (95% confidence interval, 1.55 to 3.82; *P* < 0.001) (Supplementary Table 4). Similarly, age, delirium and CCI were significant predictors for mortality (Supplementary Table 4).

### Utility of BSEEG in predicting short-term mortality

**Figure 3:**
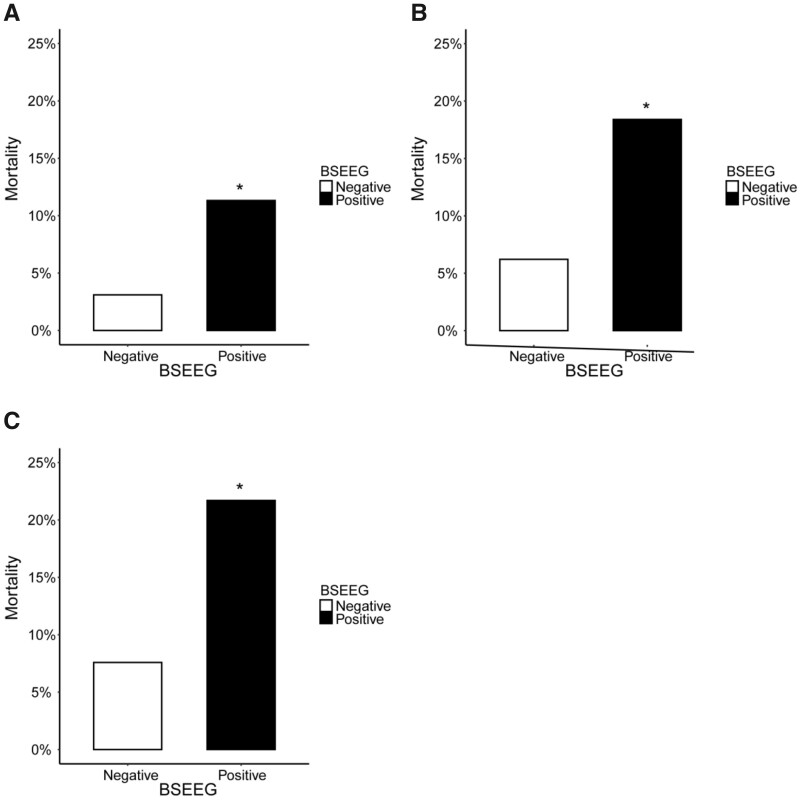
**Short-term mortality based on the BSEEG category in 502 subjects (discovery and replication cohorts).** (**A**) Thirty days; (**B**) 60 days; and (**C**) 90 days. Notes: *Relative risk was significantly higher than those in the BSEEG-negative group. BSEEG, bispectral electroencephalography.

The mortality in 30, 60 and 90 days was compared to test how soon BSEEG can differentiate mortality risk among the total 502 subjects (discovery and replication cohorts). The 30-day mortality in the BSEEG-positive group was significantly higher than that of the BSEEG-negative group (relative risk = 3.65; 95% confidence interval, 1.73–7.69; *P* < 0.001) (Fig. 3A). Similarly, 60-day mortality in the BSEEG-positive group was significantly higher than that of the BSEEG-negative group (relative risk = 2.96; 95% confidence interval, 1.74–5.03; *P* < 0.001) (Fig. 3B), as well as 90-day mortality in the BSEEG-positive group compared to the negative group (relative risk = 2.86; 95% confidence interval, 1.78–4.60; *P* < 0.001) (Fig. 3C).

Furthermore, short-term mortalities were analysed to show the difference between patients with and without dementia among the total 502 subjects (discovery and replication cohorts). The 60-day mortality with dementia in the BSEEG-positive group was significantly higher than that of the BSEEG-negative group (relative risk = 3.00; 95% confidence interval, 1.17–7.70; *P* = 0.025) as well as those without dementia (relative risk = 2.78; 95% confidence interval, 1.46–5.28; *P* = 0.001) (Supplementary Fig. 1). Similarly, the 90-day mortality in those with dementia in the BSEEG-positive group was significantly higher than those with dementia in the BSEEG-negative group (relative risk = 3.80; 95% confidence interval, 1.52–9.48; *P* = 0.002) as well as in those without dementia (relative risk = 2.39; 95% confidence interval, 1.35–4.22; *P* = 0.003) (Supplementary Fig. 1).

## Discussion

The present study shows the utility of BSEEG in predicting mortality in an independent cohort by conducting a replication study. Furthermore, the mortality in patients with dementia who showed a high BSEEG score was higher than those with dementia who showed a negative BSEEG score. The result was consistent with our hypothesis that BSEEG score can predict mortality among dementia patients. This is the first study to show the utility of BSEEG score in predicting mortality in dementia patients.

Previously, findings about the usefulness of the BSEEG had been limited to a study of delirium and mortality in 274 elderly inpatients.^9^ In the present study, we showed the utility of the BSEEG in predicting mortality with an independent cohort and a cohort in an increased sample size. Moreover, a score-dependent increase in mortality as identified by BSEEG score was replicated as shown in a previous cohort.^9^ As it is important to assess risks of outcomes including mortality in elderly inpatients to optimize intervention and care planning, numerous measures to evaluate the risk of mortality have been developed as shown below. For example, the CCI is used for predicting mortality by evaluating comorbidity.^19^ Similarly, various measures such as the Multidimensional Prognostic Index,^20^ the Elixhauser Comorbidity System^21^ and the single general self-rated health^22^ are used for predicting mortality. However, these measures mentioned above have the shared limitation of lacking biological basis. In addition to the above measures, the BSEEG score has the potential to be used for predicting mortality as an electrophysiological biomarker. We believe that the BSEEG is sensitive to detect brain signal abnormality with slow-wave characteristic to delirium or inflammatory process in brain, which is sometimes hard to capture in clinical settings. In fact, our group recently developed a pre-clinical model to assess neuroinflammation measured by BSEEG method applied to a mouse in response to systemic inflammation induced by lipopolysaccharides.^23^ The model showed clear dose-dependent increase of BSEEG proportionate to dose of lipopolysaccharides and the effect was more drastic in aged mouse compared to young mouse.^23^ We speculate that is why the BSEEG is capable to predict patients’ mortality.

In the present study, the utility of BSEEG for predicting mortality was shown for dementia patients as well. This result suggests that we may be able to predict mortality among dementia patients by using BSEEG score rather than just relying on clinical diagnoses for delirium. Although an appropriate intervention can improve outcomes of patients with delirium,^24^^,^^25^ it is well known that detection of delirium in patients with dementia is challenging.^26–29^ Therefore, detection of patients with a BSEEG-positive score followed by prompt intervention may improve patient outcomes whether or not they have dementia.

Importantly, there was a significant difference of mortality even in 30 days between BSEEG-positive and BSEEG-negative groups. According to the result of the present study, approximately one in eight who were BSEEG-positive died in 30 days, whereas one in 32 who were BSEEG-negative died in 30 days. It is important to predict short-term outcomes in elderly inpatients because their outcome may be directly related to death. In previous studies, there are significant relationships not only between a long-term mortality longer than a year and delirium,^30^^,^^31^ but also a short-term mortality within several months and delirium.^32^^,^^33^ The present finding suggests that BSEEG may be useful for predicting both a short-term and a long-term mortality in elderly inpatients. This result is consistent with the recent prospective cohort study^10^ and retrospective cohort study^11^ showing that clinical electroencephalography slowing is associated with an increased mortality. Furthermore, short-term mortalities and relative risks in BSEEG-positive patients were higher in patients with dementia compared to those without dementia. According to the results of the present study, approximately one in three with a BSEEG-positive score and dementia died in 90 days, whereas one in six with a BSEEG-positive score but without dementia. These results indicate that BSEEG may be useful for patients with dementia to predict short-term mortality.

There are several limitations of this study. First, determination of dementia status relied on chart review. Thus, there might be many cases of over- or under-diagnoses. In addition, we did not evaluate severity of dementia in the present study. A previous study showed that severity of dementia predicts mortality of patients with dementia.^34^ To overcome this limitation, we need to diagnose dementia with a more detailed approach in future study. Second, the average ages in cases with delirium and dementia, and in the BSEEG-positive group, were higher than those in controls and in the BSEEG-negative group. However, delirium and BSEEG were significant predictors for mortality according to results of the Cox proportional hazards model even after adjusting for age. Lastly, because this study was conducted only in a general hospital located in the Midwest of the USA, the results may not be generalizable. To overcome this limitation, multi-centre research is needed in the future.

Even with these limitations, this study suggests that BSEEG score can predict mortality among elderly patients in general, and even among dementia patients, as soon as 30 days after their hospital admission.

## Supplementary material

Supplementary material is available at *Brain Communications* online.

## Funding

This study was funded by research grants from National Science Foundation 1664364.

## Competing interests

G.S. is a co-founder of Predelix Medical LLC, and reports U.S. Provisional Patent Application No. 62/829411, titled ‘Prediction of patient outcomes with a novel electroencephalography device’. The other authors report no biomedical financial interests or potential conflicts of interest.

## Supplementary Material

fcab037_Supplementary_Data

## Abbreviations

BSEEG =: bispectral electroencephalography;
CCI =: Charlson Comorbidity Index
