# Supplementary material for: Mortality prediction by bispectral electroencephalography among 502 patients: its role in dementia
Source: Brain Commun. 2021 Mar 13;3(2):fcab037. doi: 10.1093/braincomms/fcab037 (PMC8204260; doi:10.1093/braincomms/fcab037)
Supplement: fcab037_Supplementary_Data [file fcab037_supplementary_data.pdf]

Supplementary Materials

Supplementary Table 1 Demographic characteristic of the replication cohort (N = 228)

Supplementary Table 2 Result of the Cox proportional hazard model (N = 228)

Supplementary Table 3 Result of the Cox proportional hazard model (N = 502)

Supplementary Table 4 Result of the Cox proportional hazard model (N = 502)

Supplementary Figure 1 Short-term Mortality Based on the Dementia and BSEEG Categories in 502 Subjects (Discovery and Replication Cohorts)

Supplementary Table 1 Demographic characteristic of the replication cohort (N = 228)

	Delirium		Dementia		Day1 BSEEG	
	Case	Control	Case	Control	Positive	Negative
n	67	161	36	192	74	154
Female, n (%)	29 (43.3)	82 (50.9)	25 (69.4) *	86 (44.8)	32 (43.2)	79 (51.3)
Age, mean (SD), (y)	72.7 (9.4)**	69.9 (9.5)	73.2 (8.8) **	70.2 (9.6)	71.6 (9.3) **	70.3 (9.6)
CCI, mean (SD)	4.3 (2.7) **	2.5 (2.9)	4.1 (3.0) **	2.8 (2.9)	3.4 (3.1) **	2.8 (2.8)

Note: * $p<0.05$ vs. Control or Negative, ** $p<0.01$ vs. Control or Negative.

Abbreviations: BSEEG, bispectral electroencephalography; CCI, Charlson Comorbidity Index.

Supplementary Table 2 Result of the Cox proportional hazard model (N = 228)

	Hazard Ratio	95% Confidence interval	p-value
BSEEG, Positive	2.82	1.33-6.00	0.007
Age	1.05	1.01-1.10	0.012
Sex, Female	1.49	0.71-3.14	0.296
CCI	1.19	1.08-1.31	<0.001
Delirium	2.14	1.01-4.54	0.048

Abbreviations: BSEEG, bispectral electroencephalography; CCI, Charlson Comorbidity Index.

Supplementary Table 3 Result of the Cox proportional hazard model (N = 502)

	Hazard Ratio	95% Confidence interval	p-value
BSEEG, Positive	2.43	1.55-3.82	<0.001
Age	1.04	1.01-1.06	0.002
Sex, Female	1.09	0.71-1.66	0.709
CCI	1.18	1.11-1.25	<0.001
Delirium	2.00	1.30-3.09	0.002

Abbreviations: BSEEG, bispectral electroencephalography; CCI, Charlson Comorbidity Index.

Supplementary Table 4 Result of the Cox proportional hazard model (N = 502)

	Hazard Ratio	95% Confidence interval	p-value
BSEEG, Positive	2.43	1.55-3.82	<0.001
Age	1.04	1.01-1.06	0.004
Sex, Female	1.07	0.70-1.65	0.747
CCI	1.18	1.11-1.25	<0.001
Delirium	1.97	1.25-3.09	0.003
Dementia	1.08	0.66-1.76	0.760

Abbreviations: BSEEG, bispectral electroencephalography; CCI, Charlson Comorbidity Index.

Supplementary Figure 1 Short-term Mortality Based on the Dementia and BSEEG Categories in 502 Subjects (Discovery and Replication Cohorts)

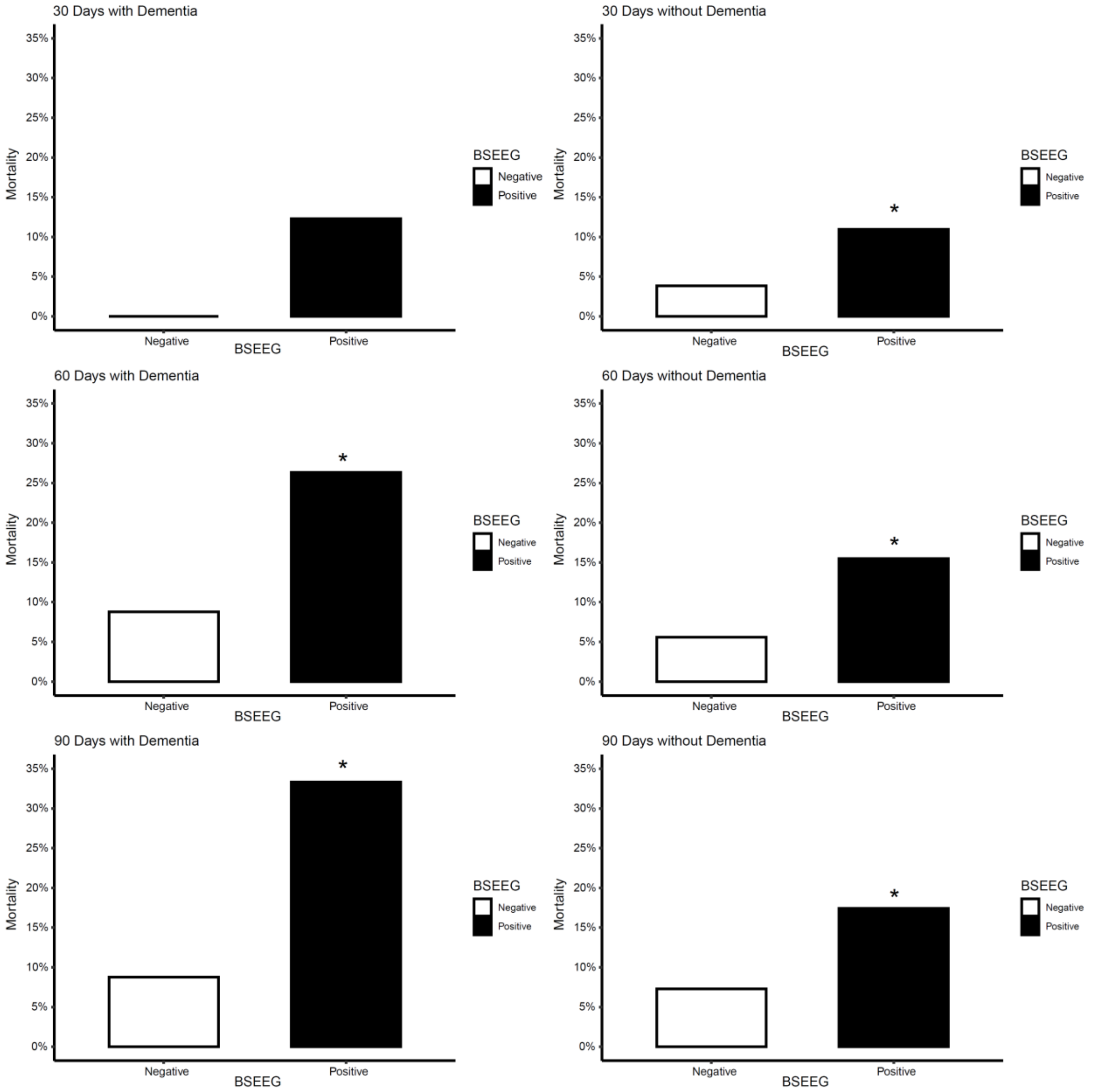

Notes: * Relative risk was significantly higher than those in the BSEEG-negative group.

Abbreviation: BSEEG, bispectral electroencephalography
